# Acupoint combination effect of Shenmen (HT 7) and Sanyinjiao (SP 6) in treating insomnia: study protocol for a randomized controlled trial

**DOI:** 10.1186/s13063-020-4170-1

**Published:** 2020-03-12

**Authors:** Pei Wu, Cisong Cheng, Xiaojun Song, Ling Yang, Dandan Deng, Zhenrong Du, Xingyu Chen, Tingting Zou, Ling Qiao, Na Li, Pingping Zhou, Li Du, Yihui Zhu

**Affiliations:** 1 Acupuncture and Tuina School/3rd Teaching Hospital, Chengdu University of Traditional Chinese Medicine, 37 Shierqiao Road, Chengdu, 610075 Sichuan China; 2 School of Basic Medicine, Chengdu University of Traditional Chinese Medicine, 1166 Liutai Avenue, Chengdu, 611137 Sichuan China; 3 Chinese Acupuncture of 1st Teaching Hospital of Chengdu University of Traditional Chinese Medicine, 39 Shierqiao Road, Chengdu, Sichuan China

**Keywords:** Insomnia, Randomized controlled trial, Study protocol, Acupoint combination, Shenmen (HT 7), Sanyinjiao (SP 6)

## Abstract

**Background:**

Insomnia is a global disease with a high incidence and acupuncture therapy is a well appropriate method to treat insomnia. Shenmen (HT 7) and Sanyinjiao (SP 6) are the acupoints most commonly used to treat insomnia. Although they can obviously relieve the clinical symptoms of insomnia, it is unclear whether they must be used together, whether the combination of two acupoints may have a synergistic or antagonistic effect, and whether there is a primary or secondary relationship between the two points in the treatment of insomnia. Further studies are needed. Therefore, in this study, we are exploring the acupoint combination effect and biological mechanism of HT 7 and SP 6 in treating insomnia.

**Methods/design:**

This will be a parallel group randomized controlled trial. The study will recruit 120 patients with insomnia randomly assigned to a control group, an electroacupuncture on HT 7 group, an electroacupuncture on SP 6 group, and an electroacupuncture on HT 7 and SP 6 group. The allocation ratio is 1:1:1:1, with 30 subjects in each group. Meanwhile, ten healthy subjects who meet the study criteria will be recruited as the healthy control group. Patients in the intervention groups will be given ten rounds of electroacupuncture stimulation on the corresponding acupoints for 2 weeks, five times per week, with 2 days of rest between the two treatment courses. Patients in the control group will also receive the same two courses of ten rounds of compensatory acupuncture therapy after a 2-week waiting period for treatment. The major outcome measures of this study include the Sleep Dysfunction Rating Scale, the Insomnia Severity Index, Epworth Sleepiness Scale, the Zung Self-Rating Anxiety Scale, and the Zung Self-Rating Depression Scale, combined with the Measure Your Medical Outcome Profile, to evaluate insomnia and the emotional state of patients with insomnia. The secondary outcome measures include sleep composition monitored by polysomnography and measurements of acetylcholine, serotonin, dopamine, norepinephrine, melatonin, gamma-aminobutyric acid, and metabolic biomarkers in serum.

**Discussion:**

In this study, we are exploring the acupoint combination effect and biological mechanism of HT 7 and SP 6 in treating insomnia, which may provide evidence for the clinical application of acupuncture and acupoint selection in the treatment of insomnia.

**Trial registration:**

Chinese Clinical Trial Registry, Chi-CTR-1800017483. Registered on 1 August 2018.

## Background

Acupoint combination is a method to improve clinical curative effects by combining two or more acupoints to achieve specific therapeutic effects [1]. It is a very important factor affecting clinical curative effect. Maximizing the efficacy of acupoint combination is the key to improving the curative effect of acupuncture and moxibustion [2].

Insomnia is a global disease with a high incidence. The incidence is above 35% in recent years [3, 4]. Insomnia is a serious deterioration of sleep quality, usually accompanied with serious residual effects during the daytime, including dizziness, headache, malaise, fatigue, anxiety, and other symptoms, which may reduce quality of life and activity efficiency, affect physical and mental health, and even cause many kinds of diseases [5].

Acupuncture has a long history and advantages in treating insomnia. According to statistics, 156 prescriptions and 90 acupuncture points for insomnia treatment are recorded in ancient books [6]. The acupoints selections for insomnia also reflect the idea of acupoint combination. Many clinical reports show that acupuncture therapy has definite curative effects on insomnia and certain advantages over conventional drugs [7, 8]. Studies have shown that Shenmen (HT 7) and Sanyinjiao (SP 6) are the two most frequently used acupoints for insomnia treatment [9–13]. What’s more, the two acupoints were the most common combination, the support degree of their simultaneous appearance was 59.40% among the 266 prescriptions reported from 1994 to 2014 [9]. From traditional Chinese medicine (TCM) theory, HT 7 is the original point of the heart meridian, with the function of nourishing heart zang and tranquilizing the mind, which are good for sleep. SP 6 belongs to the spleen channel, being the intersection of liver, kidney, and spleen channels, with the function of coordinating and reinforcing qi and blood, which helps to nourish heart zang and soothe the nerves to improve sleep. The combination of HT 7 and SP 6 has the effect of harmonizing yin and yang, calming heart and soothing the nerves, to bring peaceful sleep. It was found that electroacupuncture stimulation on HT 7 and SP 6 has good effects on the sympathetic adrenal medullary system and improves anxiety in a rat insomnia model [14, 15]

Previous studies mainly count the frequency of the selections and collocation of them, which is not enough to reflect the deeper law of acupoint combination in treating insomnia. Because the law, effect, and mechanism of it are not thoroughly studied [16], and because of the lack of experimental evidence [17], there is a certain gap between acupoint combination research and clinical application of acupuncture therapy.

Although HT 7 and SP 6 can relieve clinical symptoms and adjust neuroendocrine function of patients with insomnia, further studies are needed, such as whether they must be used together, whether the combination of two acupoints may have a synergistic or antagonistic effect, and the primary and secondary relationships between the two points in the treatment of insomnia. This study will analyze the curative effect of the combination of HT 7 and SP 6 in treating insomnia and will reveal the biological mechanism at the metabolic level. The results can provide evidence for selection of acupoints to treat insomnia and accumulate experience for exploring the effect and mechanism of acupoint combination.

## Methods/design

### Study design

**Fig. 1:**
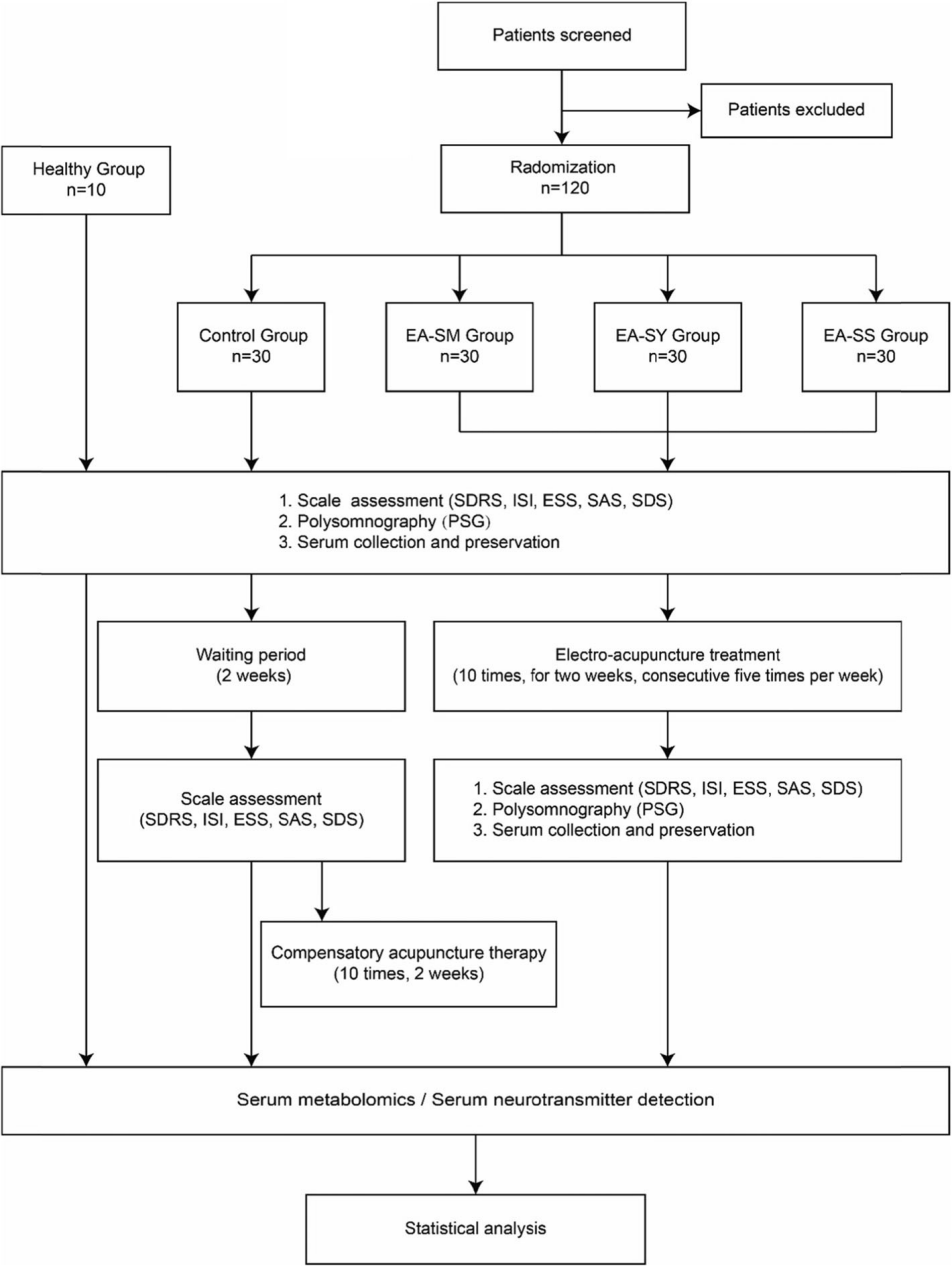
Overall schematic chart of the study. The template is a Consolidated Standards of Reporting Trials (CONSORT) 2010 flow diagram. *EA-SM Group* electroacupuncture on Shenmen (HT 7), *EA-SY Group* electroacupuncture on Sanyinjiao (SP 6), *EA-SS Group* electroacupuncture on Shenmen (HT 7) and Sanyinjiao (SP 6), *SDRS* Sleep Dysfunction Rating Scale, *ISI* Insomnia Severity Index, *ESS* Epworth Sleepiness Scale, *SAS* Zung Self-Rating Anxiety Scale, *SDS* Zung Self-Rating Depression Scale

**Fig. 2:**
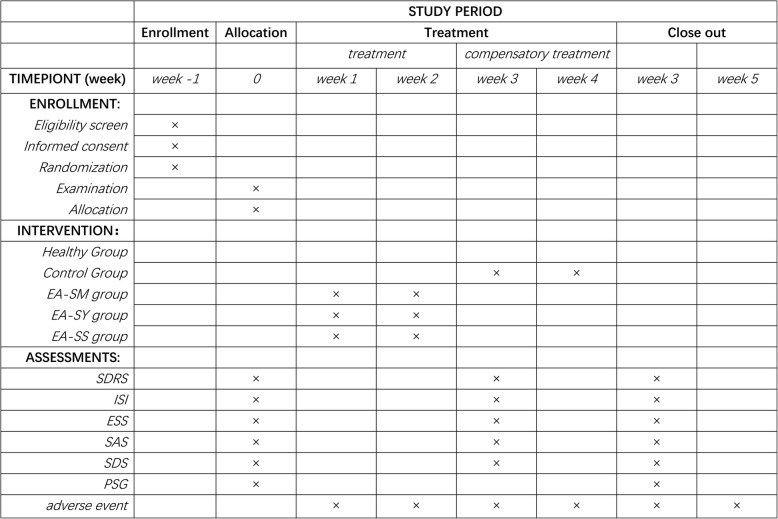
Study schedule of enrollment, interventions, and assessments. The template is derived from the Standard Protocol Items: Recommendations for Interventional Trials (SPIRIT) 2013 statement. The patients in the control group will finish the assessments and then receive compensatory acupuncture therapy at week 3 after the 2-week waiting period (week 1, week 2). *EA-SM Group* electroacupuncture on Shenmen (HT 7), *EA-SY Group* electroacupuncture on Sanyinjiao (SP 6), *EA-SS Group* electroacupuncture on Shenmen (HT 7) and Sanyinjiao (SP 6), *SDRS* Sleep Dysfunction Rating Scale, *ISI* Insomnia Severity Index, *ESS* Epworth Sleepiness Scale, *SAS* Zung Self-Rating Anxiety Scale, *SDS* Zung Self-Rating Depression Scale, *PSG* polysomnography

This will be a parallel group randomized controlled trial. The study will recruit 120 patients with insomnia from the Affiliated Hospital of Chengdu University of TCM and the Third Affiliated Hospital of Chengdu University of TCM. Meanwhile, ten healthy subjects who meet the study criteria will be recruited as the healthy control group. The patients enrolled will be randomly assigned to a control group, an electroacupuncture on HT 7 group (EA-SM group), an electroacupuncture on SP 6 group (EA-SY group), and an electroacupuncture on HT 7 and SP 6 group (EA-SS group). The allocation ratio is 1:1:1:1, with 30 subjects in each group. All patients will be asked to sign the consent form before the trial. Patients in the intervention groups will be given ten rounds of electroacupuncture stimulation on corresponding acupoints for 2 weeks, five times per week, with 2 days of rest between the two treatment courses. Patients in the control group will also receive the same two courses, ten rounds of compensatory acupuncture therapy after the 2-week waiting period for treatment. The healthy control group will not be given any intervention. The overall schematic and study design are presented in Fig. 1, and the schedule of enrollment, intervention, and assessments is shown in Fig. 2, according to the Standard Protocol Items: Recommendations for Interventional Trials (SPIRIT) 2013 statement [18].

## Participants

### Criteria for patients with insomnia

#### Inclusion criteria

We will include patients who (1) meet the diagnostic criteria for insomnia (referring to the guidelines for the diagnosis and treatment of adult insomnia in China drawn up by the Sleep Disorders Group of Chinese Neurology Society [19]), (2) are aged 20–60 years (either sex), and (3) have received a full explanation about the research and have signed the informed consent form.

#### Exclusion criteria

Participants with any of the following conditions will be excluded from this trial: (1) secondary insomnia caused by physical disease or mental disorder; (2) those who refuse acupuncture therapy; (3) insomnia during pregnancy or lactation; (4) those who have received other sleep-related treatment within 2 weeks before the study; and (5) patients with cardiovascular, pulmonary, liver, kidney, hematopoietic, or other serious primary diseases and mental diseases.

### Criteria for healthy control subjects

#### Inclusion criteria

Inclusion criteria for healthy control subjects include: (1) without any physical or mental illness; (2) routine blood examination for liver function, kidney function, electrocardiogram, blood glucose, and lipids are normal; (3) aged from 20 to 60 years old (either sex); (4) have not participated in other clinical studies recently; (5) have signed the informed consent form and volunteered to participate in this study.

#### Exclusion criteria

Subjects will be excluded if they have any of the following conditions: (1) women during menstruation, pregnancy, or lactation and (2) alcoholism or heavy smoking.

### Sample size

This study sample size is calculated on the basis of a ratio of 1:1:1:1 between the intervention groups and the control group. Though there are four study groups, the study can be regarded as a comparison between each intervention group and the control group. This study refers to the method of sample size estimation in this paper [20]. In those studies, the sample sizes are based on studies that investigated the effect of acupuncture-related treatment on insomnia using the Sleep Dysfunction Rating Scale (SDRS) as a major outcome [21, 22], and the number of participants required for each group is 30 or 31. In addition, in the study [21], the mean change in SDRS between the intervention and control groups was 2.69, and the standard deviation was estimated from the means and the standard deviations of intervention (− 14.80 ± 3.52) and control groups (− 12.11 ± 2.38). On the basis of a two-tailed alpha error of 5% and with a statistical power of 90%, the calculated sample size is based on the equation:

$$ N=\frac{2\times {\left(\frac{Z\alpha}{2}+ Z\beta \right)}^2\times {\sigma}^2}{\delta^2} $$

$$ N=\frac{2\times {\left(1.96+1.28\right)}^2\times {3.00}^2}{2.69^2}=26.11 $$

Therefore, this study’s sample size is 120, assuming a 15% dropout rate, and all 120 participants will be randomly assigned, with 30 subjects in each group.

### Randomization and allocation concealment

One hundred twenty subjects will be randomly assigned to four groups. The random numbers are generated by the computer and sealed in opaque, airtight envelopes to form a completely random distribution scheme. According to registration order, the subject management center will open the corresponding serial number envelope in sequence and assign the patients to the corresponding experimental group. The acupuncture operators will treat the patients according to the corresponding treatment plan.

### Blinding

The participants will be blinded in this trial by being treated in an isolated room. The acupuncture operators, outcome assessors, and statisticians who perform the statistical analyses will be blinded to group assignment.

### Interventions

Patients in the three intervention groups will be given ten rounds of electroacupuncture stimulation on corresponding acupoints for 2 weeks, five times per week, with 2 days of rest between the two treatment courses. Patients in the insomnia control group will also receive the same two courses comprising ten rounds of compensatory acupuncture therapy after the 2-week waiting period for treatment. The healthy control group will not be given any intervention.

#### Selection of the acupoints

1. EA-SM group: HT 7, auxiliary acupuncture point 1
2. EA-SY group: SP 6, auxiliary acupuncture point 2
3. EA-SS group: HT 7, SP 6, auxiliary acupuncture point 1, auxiliary acupuncture point 2

All the acupoints above are selected symmetrically on the left and right sides of the limbs.

#### Location of the acupoints

**Fig. 3:**
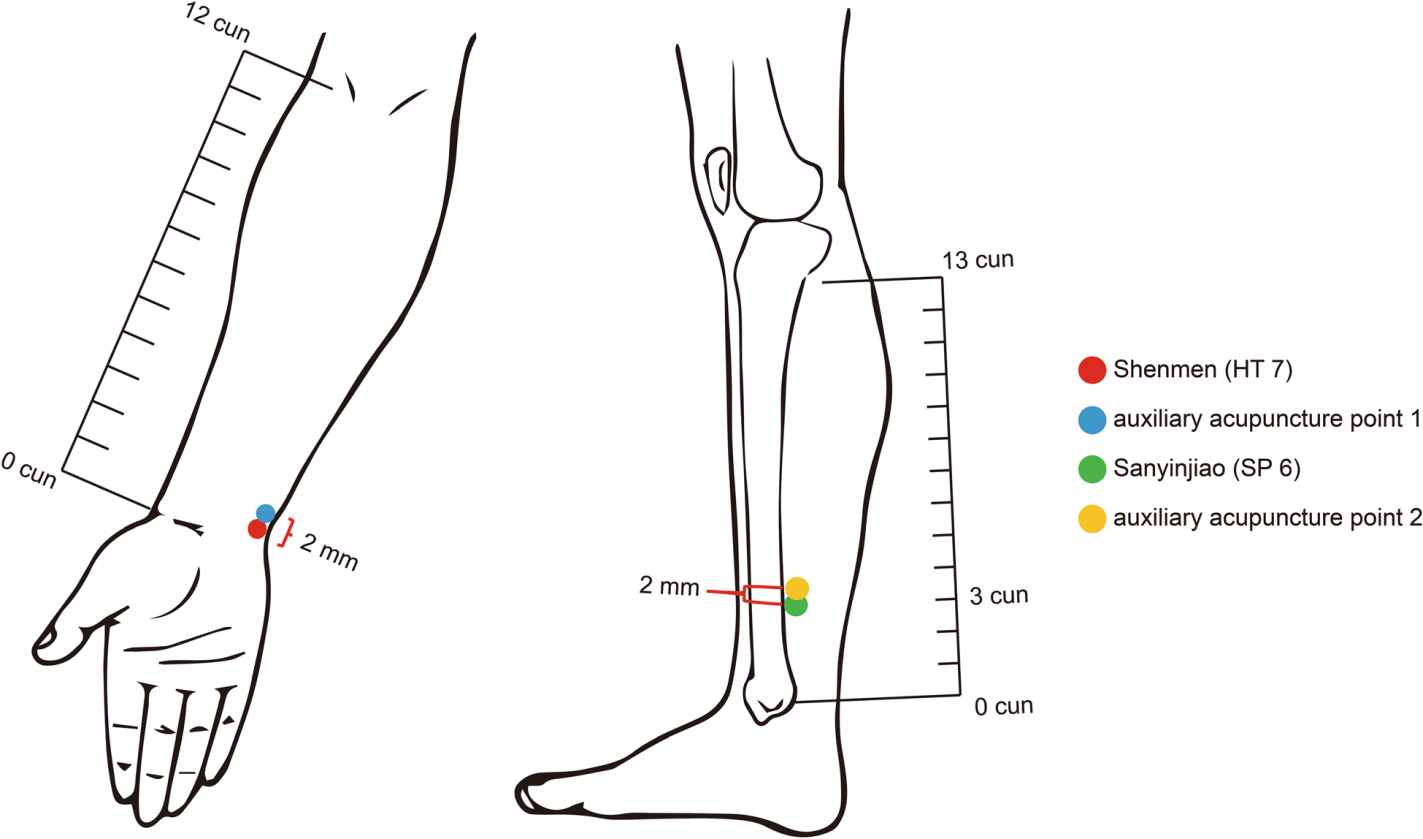
Locations of HT 7, SP 6 and auxiliary acupuncture points on one side of the body, with the same position on the other side symmetrically. Red point: HT 7; Blue piont: auxiliary acupuncture point 1; Green point: SP 6; Yellow point: auxiliary acupuncture point 2. The auxiliary acupuncture point 1 and 2 are 2mm above HT 7 and SP 6. Red and blue points are the acupoints used for EA-SM group. Green and yellow circles are the acupoints used for EA-SY group; the all circles are the acupoints used for EA-SS group

According to the National Standards of the People’s Republic of China: Name and Location of Acupoints (*GB/T 12346-2006*) to locate HT 7 and SP 6, auxiliary acupuncture point 1 and point 2 are selected according to the following references [23]:

1. HT 7: on the ulnar side of the distal volar wrist stripes and the radial margin of the flexor carpi ulnaris tendon in the anterior wrist area
2. SP 6: 3 *cun* straight up to the tip of medial malleolus, posterior border of medial tibia, on the inner side of the calf
3. Auxiliary point 1: located at 2 mm above HT 7
4. Auxiliary point 2: located at 2 mm above SP 6 Locations of HT 7, SP 6, auxiliary point 1 and 2 are as shown in the Fig. 3.

#### Therapeutic procedures

Electroacupuncture will be performed by trained acupuncture operators. A sterile acupuncture needle will be inserted into HT 7 at a depth of 0.3 to 0.5 *cun* (length 25 mm, diameter 0.25 mm; Huatuo, Suzhou Medical Supply Factory Co. Ltd., Suzhou, China), into SP 6 at a depth of 0.5 to 1 *cun* (length 40 mm, diameter 0.25 mm; Huatuo, Suzhou Medical Supply Factory Co. Ltd., Suzhou, China) with the de qi sensation (a compositional sensation including soreness, numbness, distention, and heaviness), and into the auxiliary points to a depth of 0.2 *cun* (length 13 mm, diameter 0.18 mm; Huatuo, Suzhou Medical Supply Factory Co. Ltd., Suzhou, China) without the de qi sensation. Then the operators will apply electroacupuncture stimulation (SDZ-II electroacupuncture therapy apparatus; Huatuo, Suzhou Medical Supply Factory Co. Ltd., Suzhou, China) by connecting HT 7 and the auxiliary point 1 as one pair of electrodes and connecting SP 6 and the auxiliary point 2 as another, using density-wave electrical stimulation with a frequency of 5 Hz to 25 Hz and current intensity between 1.0 mA and 1.5 mA. The output strength of electroacupuncture will be set according to the maximum tolerated intensity and the comfort feeling of each subject. The treatments will last 30 min. They will be given ten times for 2 weeks, five times per week, with 2 days of rest between the two treatment courses.

The subjects in the three intervention groups will be prohibited from receiving any other relevant treatment during the trial period. In our study, no drug combination that can improve sleep is permissible. All relevant treatments and medication compliance will be recorded in the case report form.

## Outcome measures

### Major outcome measures

The major outcome measure in this study is the SDRS, which is a quantitative assessment tool for insomnia severity based on the Chinese classification and diagnostic criteria for mental disorders (third edition), with good reliability and validity for assessing sleep quality, sleep duration, different clinical manifestations of insomnia, and the discomfort associated with insomnia [24].

### Secondary outcome measures

Secondary outcome measures of this study include the Insomnia Severity Index (ISI), the Epworth Sleepiness Scale (ESS), the Zung Self-Rating Anxiety Scale (SAS), and the Zung Self-Rating Depression Scale (SDS) to evaluate the insomnia and emotional state of patients with insomnia; the Measure Your Medical Outcome Profile to evaluate the main symptoms of the patients according to self-report; the Sleep Latency(SL), Total Sleep Time(TST), and so forth collected through polysomnography (PSG); and the quantity of acetylcholine (Ach), serotonin (5-HT), dopamine (DA), norepinephrine (NE), melatonin (MT), gamma-aminobutyric acid (GABA), and metabolic biomarkers in serum. By these measures, we hope to explore the acupoint combination effect of HT 7 and SP 6 in treating insomnia and its neurobiological and metabolic mechanisms.

## Data analysis

Statistical analysis includes the actual number of subjects, cases of shedding and exclusion, demographic and other baseline characteristics, compliance analysis, safety assessment, insomnia and emotional state scores, SL, TST, and quantity analysis of serum neurotransmitters and hormones. IBM SPSS Statistics for Windows version 19.0 software (IBM Corp., Armonk, NY, USA) will be used for statistical analysis. We will adopt univariate analysis of variance to analyze the age, height, weight, scale score, SL, TST, neurotransmitter and hormone content, and so forth, and we will use the chi-square test to analyze gender comparison and the rank-sum test to analyze the course of disease. *P* < 0.05 will be considered as the standard of statistical significance. Serum will be analyzed by liquid chromatography–mass spectrometry, and the serum metabolic biomarkers of insomnia will be screened using MetaX metabolomic data analysis software (BGI Genomics Co. Ltd., Shenzhen, China).

## Quality control and guarantee

The researchers are trained before the study, including the research process, implementing rules, criteria for inclusion and exclusion of diagnosis, treatment regimen, acupoint selection and acupuncture operation, curative effect observation, data collection and management, and adverse event reporting and recording. The trial will be terminated under the following conditions: serious adverse events, serious complications, or deterioration of condition, in which case the clinical trial should be stopped according to the judgment of the doctor, or when participants are reluctant to continue the study and ask to drop out, or upon occurrence of special physiological changes (such as pregnancy) during the study period that make it inappropriate to continue. A study coordination and management team is set up to take charge of the management and quality control of the test program and supervise the research process, ensuring that the research data recording and reporting are correct, complete, and consistent with the original data. The scale evaluation is completed by particular persons. PSG is conducted by professionals according to professional testing procedures in the Sleep Medicine Center of West China Hospital of Sichuan University. Serum metabolomics are tested and analyzed by BGI Genomics Co. Ltd. Neurotransmitters and hormones are detected in strict accordance with the kit instructions. When the subjects drop out, they will be interviewed face to face, followed up by phone or mail and so forth to ask for reasons, record the time of the last treatment, and complete the evaluation items as far as possible. For patients who withdraw from the study due to adverse reactions, ineffective treatment, or other changes in their conditions, corresponding treatment measures shall be taken according to the actual conditions of the subjects. Patients receiving more than half of the treatment course among those who have been ranked with random numbers will be included in the statistical analysis. All dropouts will be analyzd according to intention-to-treat principle after the trial.

## Recording and handling of adverse events

The researchers explain to the patient and ask the patient and his/her family to truthfully reflect the changes in their condition after treatment and will avoid leading questions. When observing the curative effect, researchers should also pay attention to the adverse reaction. Regardless of the correlation between adverse reactions and events and this study method, the occurrence time, symptoms, signs, extent, duration, treatment method, process, and results should be recorded in detail. When adverse events are found, necessary treatment should be taken until the patients are in stable condition.

## Discussion

This study is a parallel group randomized controlled trial exploring the effects and mechanisms of acupoint combination of HT 7 and SP 6 for treating insomnia. Multiple scales are used to comprehensively evaluate patients’ sleep quality at night and their energy and emotional state during daytime, including SDRS, ISI, ESS, SAS, and SDS. PSG is a common method of neural electrophysiological monitoring in sleep research that is often used for objective analysis of sleep. It not only can analyze the sleep structure and sleep phase of patients and reflect the degree of insomnia but also analyze the corresponding changes in heart, breathing, and muscle activity while sleeping. This study observes the effects of a single use of HT 7 or SP 6 and the joint use of the two acupoints for treating insomnia from multiple perspectives, according to subjective self-perception and objective sleep structure of patients with insomnia by using multiple scales and PSG.

Multiple nerve centers and neurotransmitters take part in the regulation of sleep. Studies have shown that the human body clock system (e.g., suprachiasmatic nucleus) periodically stimulates the sleep-inducing area and the wake-inducing area and inhibits or facilitates the cerebral cortex through the ascending inhibitory system or activation system, resulting in sleep or wakefulness [25]. Ach, 5-HT, DA, and NE may play a crucial part in sleep regulation in the sleep inhibitory or activation systems [26]. In addition, MT and GABA also play an important role. So, this study will quantify the Ach, 5-HT, DA, NE, MT, and GABA in serum of subjects in the healthy control group, the control group, and the three intervention groups using enzyme-linked immunosorbent assays to explore the neurotransmitter regulatory mechanism of the effect that improves sleep by acupoint combination.

Metabolomics, guided by the theory of system biology, monitors the changes of endogenous metabolites of organisms and directly reflects the body’s response to external pathophysiological stimulation and intervention from the perspective of the whole system. It depends on spectral research and pattern recognition methods with high throughput, high sensitivity, and high accuracy. We hypothesize that the tranquilizing effect (which means the effect to improve sleep) produced by acupoint combination may be the overall effect of the integrated action of multiple links, channels, and targets in the human body. Metabolomics emphasizes the study of human beings as a complete system, coinciding with the whole view of TCM acupuncture theory. This technique has also been used to carry out research on insomnia. A study explored the mechanism of SuanZaoRen (*Ziziphi spinosae semen*) decoction to improve the sleep of rats with insomnia, screened 11 potential biomarkers related to insomnia, and obtained 8 potential metabolic pathways [27] based on ^1^H nuclear magnetic resonance metabolomics. Another study analyzed the changes of brain metabolism in mice after injection of ginseng glycoproteins by metabolomics [28]. Therefore, this study will adopt the metabolomic research method to compare the changes of endogenous metabolites before and after acupuncture intervention in different groups to find the differences in their metabolic profiles and possible related metabolic pathways, and these differential metabolic markers may be associated with insomnia. We will observe the internal mechanism of acupoint combination efficacy from a holistic, systematic, and comprehensive perspective. This can provide ideas and evidence for exploring the mechanism of acupuncture therapy on insomnia.

The study also has some limitations. In this study, the serum metabolites of the healthy control group and patients with insomnia are compared by untargeted metabolomic analysis, which is a qualitative analytic method. The possible differential metabolites can only be analyzed qualitatively but not quantitatively. The study is expected to provide a basis and ideas for further research. Further qualitative and quantitative verification can be carried out by targeting metabolomics in the future. Despite the limitations of this study, its results can provide research evidence for the study of acupoint combination, which is of great significance for the promotion and application of acupoint combination in the treatment of insomnia and has clinical value.

## Trial status

The trial is currently in the participant recruitment stage. The trial began recruitment on 12 December 2016. So far, 115 participants have been recruited, and 115 participants have completed the intervention. The recruitment is expected to be completed on 31 March 2020.

## Supplementary information

**Additional file 1.** SPIRIT 2013 Checklist: Recommended items to address in a clinical trial protocol and related documents*.

## Data Availability

Not applicable.

## Abbreviations

5-HT: Serotonin
Ach: Acetylcholine
CONSORT: Consolidated Standards of Reporting Trials
DA: Dopamine
EA-SM: Electroacupuncture on Shenmen (HT 7) group
EA-SS: Electroacupuncture on Shenmen (HT 7) and Sanyinjiao (SP 6) group
EA-SY: Electroacupuncture on Sanyinjiao (SP 6) group
ESS: Epworth Sleepiness Scale
GABA: Gamma-aminobutyric acid
ISI: Insomnia Severity Index
MT: Melatonin
NE: Norepinephrine
PSG: Polysomnography
SAS: Zung Self-Rating Anxiety Scale
SDRS: Sleep Dysfunction Rating Scale
SDS: Zung Self-Rating Depression Scale
SL: Sleep Latency
SPIRIT: Standard Protocol Items: Recommendations for Interventional Trials 2013 statement
TCM: Traditional Chinese medicine
TST: Total Sleep Time
